# Optimizing the Periconception Lifestyle of Women With Overweight Using a Blended Personalized Care Intervention Combining eHealth and Face-to-face Counseling (eFUSE): Protocol for a Randomized Controlled Trial

**DOI:** 10.2196/28600

**Published:** 2021-09-03

**Authors:** Melissa van der Windt, Sam Schoenmakers, Sten Willemsen, Lenie van Rossem, Régine Steegers-Theunissen

**Affiliations:** 1 Department of Obstetrics and Gynecology Erasmus MC, University Medical Center Rotterdam Netherlands

**Keywords:** eHealth, periconception period, lifestyle intervention, maternal and child health, pregnancy, birth outcomes, healthy lifestyle, psychotherapy, obesity, randomized controlled trial, behavior change

## Abstract

**Background:**

Maternal overweight has a substantial impact on reproductive, maternal, pregnancy, and neonatal outcomes with long-term and transgenerational health consequences. Interventions that aim to optimize periconception maternal lifestyle can improve maternal and fetal health during pregnancy and throughout the life course. However, it remains difficult to change and adopt adequate lifestyle behaviors. We hypothesize that additional psychological therapy targeting cognitive and affective factors substantially contribute to the effectiveness of these interventions.

**Objective:**

The proposed study aims to examine the feasibility and effectiveness of a blended personalized periconception lifestyle care intervention with additional psychological therapy aimed at women with a BMI≥25 and who are contemplating pregnancy or are already pregnant (≤12 weeks) in reducing inadequate lifestyle behaviors and improving early and late pregnancy outcome.

**Methods:**

The eHealth and Face-to-face Counseling (eFUSE) study follows a single-center two-arm randomized controlled trial design at the Erasmus MC, University Medical Center, with a multicenter regional referral. The female patients with overweight (BMI≥25), together with their male partner, will be stratified by pregnancy status (preconception vs pregnant) and randomized to receive either the blended personalized periconception lifestyle care intervention with additional psychological therapy (n=313) or usual care (n=313). The primary outcome is a change in the lifestyle risk score (between baseline and 24 weeks) between the randomization arms (difference in differences). Secondary outcomes include measurements defined as most relevant by the International Consortium for Health Outcomes Measurement, including behavioral determinants, patient satisfaction, provider feasibility, and maternal pregnancy and neonatal complications.

**Results:**

The study will be open for recruitment from Fall 2021 onward. Data collection is expected to be completed by the beginning of 2023, and the results are expected to be published by Fall 2023.

**Conclusions:**

This study will evaluate the feasibility and effectiveness of a blended periconception lifestyle intervention with additional psychological therapy, aimed at women with a BMI≥25. Positive results of this innovative care approach will be used for implementation in routine medical care of all women with overweight, with the ultimate aim to improve clinical outcomes of these high-risk pregnancies.

**Trial Registration:**

Netherlands Trial Register NL9264; https://www.trialregister.nl/trial/9264

**International Registered Report Identifier (IRRID):**

PRR1-10.2196/28600

## Introduction

Overweight is still a pressing health issue in the general population. During the reproductive period, maternal overweight has a significant impact on fertility, pregnancy, and neonatal outcome with long-term and transgenerational health consequences [1,2]. All these complications have been reported to increase health care use and medical expenditures [3].

Lifestyle interventions that target poor periconception maternal lifestyle behaviors have the potential to improve the chances of pregnancy and uncomplicated outcome. From 2006, we developed and tested the eHealth lifestyle coaching program Smarter Pregnancy in a large survey and randomized controlled trials (RCTs), including a general population cohort and a subfertile cohort of couples receiving either in vitro fertilization or intracytoplasmic sperm injection [4,5]. These studies demonstrated that Smarter Pregnancy is an effective, and cost-effective intervention [6]. At the same time, a periconception lifestyle counseling clinic Healthy Pregnancy and a comparable blended care clinic including the combination of face-to-face counseling and the eHealth coaching program Smarter Pregnancy was developed and evaluated [7,8]. The latter approach showed a high compliance rate; participants increased vegetable, fruit, and folic acid supplement intake; and lowered alcohol and tobacco use [8]. However, the effectiveness in women with overweight is still limited. Over the years, several other lifestyle interventions for pregnant or prepregnant women with overweight have been tested, but most interventions fail to reach clinically meaningful results [9].

Negative cognitive and affective factors, low self-efficacy, and food cravings are strongly associated with poor lifestyle behavior [10]. These factors can effectively be targeted by psychological therapies such as cognitive behavioral therapy, mindfulness, and acceptance and commitment therapy [11]. Psychological therapies provide skills to improve individual women’s ability to self-monitor their diet, learn impulse control techniques and behavioral modification strategies such as chewing slowly and taking time to enjoy food [11,12]. Especially for women with overweight and even obesity, psychological therapies are proven to be effective treatment modalities for targeting poor lifestyle behaviors [12-14]. However, to the best of our knowledge, face-to-face counseling and an eHealth program have never been combined with psychological therapies to reach the full potential of a periconception lifestyle intervention for women with overweight and their partner.

Therefore, we are initiating an RCT to study whether a blended personalized periconception lifestyle care intervention with supporting psychological therapy aimed at women with overweight or obesity who are contemplating pregnancy or are already pregnant (≤12 weeks) can significantly change and maintain adequate lifestyle behaviors.

## Methods

### Overview

The eHealth and Face-to-face Counseling (eFUSE) study follows a two-arm RCT design in a tertiary health care center, with multicenter regional referral, which is embedded in the Rotterdam periconception cohort (Predict study) [15]. The Predict study is an ongoing prospective tertiary hospital-based cohort embedded in the outpatient clinic of the Department of Obstetrics and Gynecology of the Erasmus MC, University Medical Center Rotterdam, the Netherlands. Patients included in the Predict study with overweight (BMI≥25 kg/m^2^) are eligible for inclusion in the eFUSE study. Patients included in the eFUSE study will be stratified by pregnancy status (preconception vs pregnant) and randomized to receive either blended personalized periconception lifestyle care with additional psychological therapy or usual care.

### Objective

The aim of this trial is to study if a blended personalized periconception lifestyle care approach aimed at women with a BMI≥25 who are contemplating pregnancy or already pregnant (≤12 weeks) can significantly reduce the lifestyle risk score (LRS).

### Participants

Patient couples, of whom the woman has a BMI≥25, are invited to participate in this RCT during their first visit to the outpatient antenatal clinic of the Erasmus MC, University Medical Center Rotterdam, Rotterdam, the Netherlands or one of the secondary hospitals, after which they will be referred and counseled for study participation. The first visit can either be a preconception consultation or the first antenatal session (≤12 weeks of gestation). Patient couples will be screened for eligibility by their physician when the woman is in reproductive age (18-45 years), is contemplating pregnancy or is pregnant (≤12 weeks), visits the outpatient antenatal clinic, and has overweight (BMI≥25). Exclusion criteria are multiple pregnancy, history of bariatric surgery, insufficient knowledge of Dutch language, fetal anomalies, or inability to provide informed consent. Women can be included as a single participant if the partner does not participate. When participating, the partner will attend the first face-to-face lifestyle counseling session and will have access to the online lifestyle coaching platform Smarter Pregnancy.

### Intervention

Our blended personalized periconception lifestyle care intervention with additional psychological therapy consists of:

- Three face-to-face lifestyle counseling sessions, provided by trained eFUSE counselors, as previously practiced in the proven effective outpatient antenatal clinic Healthy Pregnancy [7]. Both the counseling service and the eHealth platform are based on behavioral change theories [16-18] and best practices obtained from stakeholders in the field [7,19,20]. Psychological techniques such as motivational interviewing, elements of cognitive behavioral therapy, acceptance and commitment therapy, and mindfulness will be used to support the patient couples toward a significant and sustainable lifestyle change [21,22]. All aspects offered in the blended care intervention are personalized to the individual patient couple, based on the results of the risk assessment and lifestyle questionnaires filled out on the eHealth platform at the first visit.
- The periconception eHealth lifestyle coaching platform Smarter Pregnancy, providing personalized lifestyle risk assessment and coaching via the evidence-based eHealth intervention (www.slimmerzwanger.nl or www.smarterpregnancy.co.uk) [4,5]. Additionally, all participants will be encouraged to download and frequently use the mobile app Headspace, which provides guided meditation resources online [23].

Women allocated to the intervention arm will also be provided with standard care and routine antenatal visits according to local and Dutch guidelines [24].

#### First Counseling Session

The first counseling session takes place in week 1 of the blended personalized periconception lifestyle care intervention and will be attended by both the woman and male partner. The aim of the first counseling is twofold: first, to raise awareness on the personal lifestyle-associated risk factors for fertility and pregnancy and, second, to facilitate the ongoing provision of tailored lifestyle counseling. In the first session, together with the counselor, participants will be asked to formulate up to three lifestyle behavior goals and develop a plan to reach these goals (identifying thresholds and positive incentives).

#### Second and Third Counseling Sessions

As part of the Smarter Pregnancy coaching program, every 6 weeks a digital questionnaire on lifestyle behaviors will be filled out by the participants. During the second (in weeks 4-6 of the blended personalized periconception lifestyle care intervention) and the third counseling sessions (in weeks 16-18 of the blended personalized periconception lifestyle care intervention), results of the questionnaire will be discussed, personal lifestyle goals will be reviewed, progress of lifestyle change will be discussed, and motivational interviewing will be used to support further (sustainment of) behavior change. We expect that the effects of the face-to-face and eHealth components will reinforce each other. The second and third counseling sessions are only mandatory for the woman and her partner is welcome to attend.

During the second and third counseling sessions at the outpatient clinic Healthy Pregnancy, a functional analysis of behavior will be carried out using a situation-organism-reaction-consequences scheme, relaxation techniques will be educated, the automatic thoughts worksheet will be filled out, and the five-factor model will be discussed (Multimedia Appendices 1-3) [25-27]. These components of cognitive behavioral therapy, acceptance and commitment therapy, and mindfulness will teach the women and their partner skills to achieve and maintain long-term lifestyle change.

**Figure 1:**
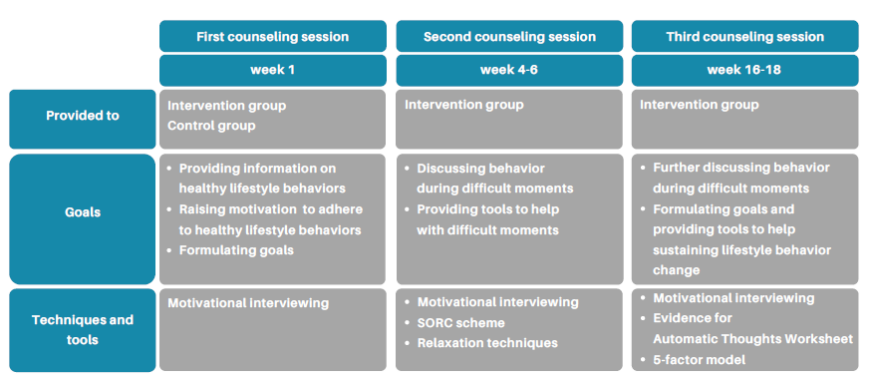
Overview of counseling sessions. SORC: situation-organism-reaction-consequences.

After the third, which is also the final lifestyle counseling session, participants will be informed that they can continue to use the eHealth platform until week 24 of the blended personalized periconception lifestyle care intervention. Participants are also informed about the possibilities to continue lifestyle counseling and support after the study period ends, which will be their own responsibility. An overview of counseling sessions and the goals and techniques used is depicted in Figure 1.

### Usual Care

Women with BMI≥25 allocated to the control group will be provided with standard care, which consists of one standard face-to-face lifestyle counseling session at the outpatient clinic Healthy Pregnancy, access to the online coaching program Smarter Pregnancy, and routine antenatal visits according to local and Dutch guidelines (NVOG protocol).

### Trial Flow

**Figure 2:**
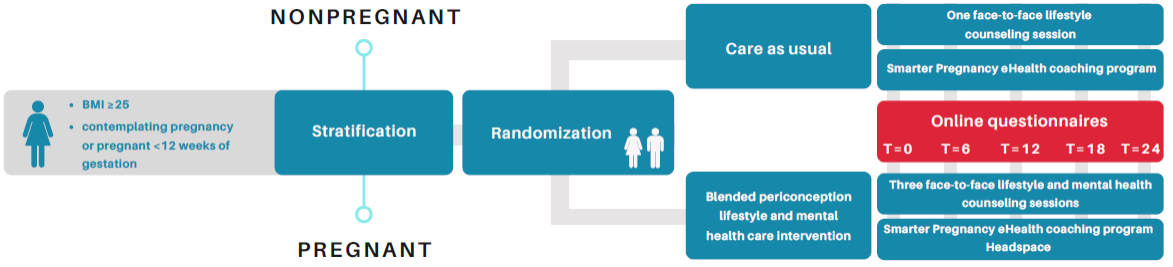
Trial flow of the eHealth and Face-to-face Counseling study.

The trial flow is visualized in Figure 2.

### Outcomes

The main outcome measure is the difference in the change of the LRS (measured in week 0 and week 24 of the blended personalized periconception lifestyle care intervention) between the intervention group and the control group (difference in differences) [28]. The LRS is a locally developed composite score of lifestyle behaviors, including vegetable and fruit intake, folic acid supplement use (only for women), not smoking, and not consuming alcohol. The vegetable and fruit intake will be subdivided into risk scores of 0, 1.5, and 3, where 0 represents an adequate daily intake (≥200 g of vegetables, ≥2 pieces of fruit). A score of 1.5 represents a nearly adequate intake (150-200 g of vegetables, 1.5-2 pieces of fruit). A score of 3 represents an inadequate daily intake (<150 g of vegetables, <1.5 pieces of fruit). Folic acid supplement use is considered to be adequate (score 0) or inadequate (score 3) when the recommended dose of 400 μg per day is either met or not. For male participants, folic acid supplement use will not be taken into account, since there are no widely supported recommendations concerning folic acid supplement use for men. The risk score for smoking will be based on average daily use: no smoking (score 0) and daily smoking of 1 to 5 (score 1), 6 to 14 (score 3), or ≥15 (score 6) cigarettes. Risk scores for alcohol consumption will be based on average weekly use: no alcohol use (score 0) and 1 to 7 (score 1), 8 to 14 (score 2), or ≥15 (score 3) alcoholic beverages (glasses) per week. The LRS ranges from 0 to 18 for women and 0 to 15 for men, and will be monitored via online questionnaires in weeks 0, 6, 12, and 24 of the online coaching program and calculated as the sum of the scores of all lifestyle behaviors.

Secondary outcome measures comprise several questionnaires, in line with the International Consortium for Health Outcomes Measurement recommendations [29]:

- *Determinants of lifestyle behavior* (eg, attitude, action control, self-efficacy, intention, and motivation) using the validated Determinants of Lifestyle Behavior Questionnaire [30], measured at weeks 0, 6, 18, and 24
- *Patient satisfaction* measured using Six Simple Questions [31] at weeks 12 and 24
- *Provider feasibility* using a 5-point Likert scale 6 and 12 months after the start of this study
- *Health status* measured using the validated EQ-5D questionnaire [32] at weeks 12, 18, and 24
- *Health-related costs*: intramural medical costs (hospital registration), iMCQ [33] (extramural medical costs), and iPCQ [34] (productivity loss)
- *Maternal pregnancy complications* (including pregnancy-induced hypertension, pre-eclampsia, HELLP, and gestational diabetes) and *neonatal outcome* (including birth weight, small for gestational age, large for gestational age, and preterm birth) retrieved from medical files

### Randomization and Allocation

Eligible consenting participants will be randomly assigned either to the intervention group or control group. The participant assignment protocol will be based on a ratio of 1:1. We will use software where randomization will be done by a predefined scheme for allocation concealment. Stratification will be made by pregnancy status (preconception vs pregnant), followed by random allocation of patients. Because of the automatic nature of the allocation process, direct exposure to the treatment allocation process by any members of the research team is eliminated.

### Data Collection

Via a lifestyle questionnaire integrated in the online coaching program Smarter Pregnancy, a baseline screening at week 0 and follow-up screening at weeks 6, 12, 18, and 24 of the program will be performed. The follow-up screening will be used to monitor the change in lifestyle components. The lifestyle questionnaire will comprise of questions regarding vegetable and fruit intake, folic acid supplementation use, tobacco use, and alcohol consumption. Participants will also be asked whether they have downloaded the Headspace app and how frequently they have used it. The additional questionnaires, concerning determinants of lifestyle behavior, patient satisfaction, and health status, will be sent to study participants.

### Sample Size Calculation

The primary outcome of this trial is the change in the LRS (between baseline and at 24 weeks) between the randomization arms (difference in differences). Based on an earlier study, we found that the SD of the change score was 1.99 [4]. We aim for a difference in change score between the arms of 0.5. To detect a difference of this size and using a significance level of .05, we can reach a statistical power of 0.80 when we have 500 women. To allow for a 20% drop out, we will include a total of 626 women, 313 in the intervention arm and 313 women in the control arm as calculated by a *t* test. This sample size calculation is based on a *t* test using unadjusted data of a survey. The actual analysis that will be done in the RCT will be less subjected to confounding factors due to the study design and analysis.

### Data Analysis

A CONSORT flowchart will be built to visualize study and participant flow in each group (intervention and control group)[35]. SPSS Statistics for Windows (IBM Corp) and R for Windows (R Foundation for Statistical Computing) will be used. Descriptive statistics for baseline values will be calculated, and comparison between the two groups will be performed by using an independent sample *t* test for continuous variables and a chi-square test for categorical variables. Linear regression analyses adjusted for baseline characteristics will be carried out to see whether the LRS differ significantly between the intervention and the control group. Subgroup analyses will be performed according to pregnancy status. All analyses will be done according to the intention-to-treat principles, and a *P* value<.05 will be considered statistically significant.

### Data Management

All researchers involved in the study will be qualified physicians, and they will follow a course in which the organization of clinical studies is taught. According to the original observation records, researchers will complete the case report forms in an accurate and timely manner. All documents will be properly classified, preserved under confidential conditions, and archived. Statistical analysis of the data will be carried out on a pseudonymized data set. Only the data manager will have access to the key file, in which the pseudonymized data is linked to the personal data.

### Safety Monitoring and Reporting

This study will have monitoring for quality and regulatory compliance and adheres to the Dutch Data Protection Act. The data being stored will be encrypted. All communication with the participants will take place within the secure online interface that requires an electronic identification.

### Ethical Approval and Dissemination

The trial will be conducted in accordance with the principles of the Declaration of Helsinki (2013 version) and will fully comply with the SPIRIT (Standard Protocol Items: Recommendations for Interventional Trials) reporting guidelines [36]. This study was approved by the Committee of Medical Ethics of the Erasmus Medical Center, Rotterdam (MEC-2020-0113) and registered in the Dutch Trial Registry (NL9264). Written informed consent from each participant will be obtained. The results will be published in peer-reviewed journals to ensure widespread dissemination.

## Results

Inclusion will start from Fall 2021 onward at the Erasmus MC, University Medical Center, Rotterdam, the Netherlands, while secondary hospitals can refer patients for inclusion. Data collection is expected to be completed by the beginning of 2023, and the results are expected to be published by Fall 2023.

## Discussion

### Overview and Expectations

The eFUSE study will evaluate an innovative blended lifestyle care intervention combined with psychological therapy to improve periconception lifestyle behaviors in women with overweight using an RCT. The intervention group will be provided with a blended care approach, including three face-to-face counseling sessions, and access to the periconception eHealth lifestyle care platform Smarter Pregnancy and mobile health app Headspace. In the face-to-face counseling sessions, motivational interviewing will be used and components of cognitive behavioral therapy, acceptance and commitment therapy, and mindfulness will be practiced. The control group will receive standard care, which comprises of one face-to-face counseling session and access to the periconception eHealth lifestyle care platform Smarter Pregnancy. The addition of components of several psychological therapies to a proven effective blended care approach is new and might result in a measure to improve parental lifestyle behaviors before and during pregnancy. We hypothesize that the two additional face-to-face counseling sessions, in which several psychological techniques will be practiced, will support the participating patient couples toward a significant and more sustainable lifestyle change. Moreover, we expect that the effects of the face-to-face sessions and eHealth program reinforce each other. By choosing a proximal primary outcome measure, namely, the LRS, we aim to assess the effects directly influenced by the intervention so that the results can be clearly deduced from the content of our approach.

### Strengths and Limitations

This is the first study that will evaluate a blended periconception lifestyle intervention with additional psychological therapy aimed at women with a BMI≥25 in a randomized controlled design. All aspects offered in the blended care approach are personalized to the individual patient couple. Furthermore, both the periconception eHealth lifestyle platform Smarter Pregnancy and the outpatient clinic Healthy Pregnancy have been proven effective before [4,5,7,8]. A possible limitation of this study is that it is hard to unravel which part of this blended periconception lifestyle intervention with mental health components affects lifestyle behaviors and which elements contribute the most.

### Conclusion

This study will evaluate a blended periconception lifestyle care intervention with additional psychological therapy aimed at women with a BMI≥25 and their partner. If effective at improving lifestyle behaviors, this approach could be an important measure for all women with overweight or obesity with the potential to improve clinical outcomes for these women who are in the preconception period and are at high risk for pregnancy complications.

## Appendix

Multimedia Appendix 1 Situation-organism-reaction-consequences scheme.

Multimedia Appendix 2 Automatic thought sheet.

Multimedia Appendix 3 Five-factor model.

## Abbreviations

eFUSE: eHealth and Face-to-face Counseling
LRS: lifestyle risk score
PRISMA: Preferred Reporting Items for Systematic Reviews and Meta-Analyses
RCT: randomized controlled trial
SPIRIT: Standard Protocol Items: Recommendations for Interventional Trials
