# Supplementary material for: Optimizing the Periconception Lifestyle of Women With Overweight Using a Blended Personalized Care Intervention Combining eHealth and Face-to-face Counseling (eFUSE): Protocol for a Randomized Controlled Trial
Source: JMIR Res Protoc. 2021 Sep 3;10(9):e28600. doi: 10.2196/28600 (PMC8449297; doi:10.2196/28600)
Supplement: Multimedia Appendix 2 [file resprot_v10i9e28600_app2.docx]

Supplemental file 2. Automatic thought sheet

|  | **Situation** | **Initial thought** | **Negative thinking** | **Evidence for this thinking** | **Alternative thinking** |
| --- | --- | --- | --- | --- | --- |
| **1.** |  |  |  |  |  |
| **2.** |  |  |  |  |  |
