# Supplementary material for: Optimizing the Periconception Lifestyle of Women With Overweight Using a Blended Personalized Care Intervention Combining eHealth and Face-to-face Counseling (eFUSE): Protocol for a Randomized Controlled Trial
Source: JMIR Res Protoc. 2021 Sep 3;10(9):e28600. doi: 10.2196/28600 (PMC8449297; doi:10.2196/28600)
Supplement: Multimedia Appendix 3 [file resprot_v10i9e28600_app3.docx]

Supplemental file 3. Five factor model

Behavior

Emotions

Physical reactions

Altered thinking

Life situation
